# Therapeutic Administration of Oxcarbazepine Saves Cerebellar Purkinje Cells from Ischemia and Reperfusion Injury Induced by Cardiac Arrest through Attenuation of Oxidative Stress

**DOI:** 10.3390/antiox11122450

**Published:** 2022-12-12

**Authors:** Yang Hee Kim, Tae-Kyeong Lee, Jae-Chul Lee, Dae Won Kim, Seongkweon Hong, Jun Hwi Cho, Myoung Cheol Shin, Soo Young Choi, Moo-Ho Won, Il Jun Kang

**Affiliations:** 1 Department of Surgery, Kangwon National University Hospital, School of Medicine, Kangwon National University, Chuncheon, Gangwon 24289, Republic of Korea; 2 Department of Food Science and Nutrition, Hallym University, Chuncheon, Gangwon 24252, Republic of Korea; 3 Department of Neurobiology, School of Medicine, Kangwon National University, Chuncheon, Gangwon 24341, Republic of Korea; 4 Department of Biochemistry and Molecular Biology, Research Institute of Oral Sciences, College of Dentistry, Gangnung-Wonju National University, Gangneung, Gangwon 25457, Republic of Korea; 5 Department of Emergency Medicine, Kangwon National University Hospital, School of Medicine, Kangwon National University, Chuncheon, Gangwon 24289, Republic of Korea; 6 Department of Biomedical Science, Research Institute for Bioscience and Biotechnology, Hallym University, Chuncheon, Gangwon 24252, Republic of Korea

**Keywords:** asphyxial cardiac arrest, cerebellar Purkinje cells, oxcarbazepine, oxidative stress, antioxidant enzymes

## Abstract

Research reports using animal models of ischemic insults have demonstrated that oxcarbazepine (a carbamazepine analog: one of the anticonvulsant compounds) extends neuroprotective effects against cerebral or forebrain injury induced by ischemia and reperfusion. However, research on protective effects against ischemia and reperfusion cerebellar injury induced by cardiac arrest (CA) and the return of spontaneous circulation has been poor. Rats were assigned to four groups as follows: (Groups 1 and 2) sham asphyxial CA and vehicle- or oxcarbazepine-treated, and (Groups 3 and 4) CA and vehicle- or oxcarbazepine-treated. Vehicle (0.3% dimethyl sulfoxide/saline) or oxcarbazepine (200 mg/kg) was administered intravenously ten minutes after the return of spontaneous circulation. In this study, CA was induced by asphyxia using vecuronium bromide (2 mg/kg). We conducted immunohistochemistry for calbindin D-28kDa and Fluoro-Jade B histofluorescence to examine Purkinje cell death induced by CA. In addition, immunohistochemistry for 4-hydroxy-2-nonenal (4HNE) was carried out to investigate CA-induced oxidative stress, and immunohistochemistry for Cu, Zn-superoxide dismutase (SOD1) and Mn-superoxide dismutase (SOD2) was performed to examine changes in endogenous antioxidant enzymes. Oxcarbazepine treatment after CA significantly increased the survival rate and improved neurological deficit when compared with vehicle-treated rats with CA (survival rates ≥ 63.6 versus 6.5%), showing that oxcarbazepine treatment dramatically protected cerebellar Purkinje cells from ischemia and reperfusion injury induced by CA. The salvation of the Purkinje cells from ischemic injury by oxcarbazepine treatment paralleled a dramatic reduction in 4HNE (an end-product of lipid peroxidation) and increased or maintained the endogenous antioxidant enzymes (SOD1 and SOD2). In brief, this study shows that therapeutic treatment with oxcarbazepine after CA apparently saved cerebellar neurons (Purkinje cells) from CA-induced neuronal death by attenuating oxidative stress and suggests that oxcarbazepine can be utilized as a therapeutic medicine for ischemia and reperfusion brain (cerebellar) injury induced by CA.

## 1. Introduction

The cerebellum plays important roles in motor control and may be involved in cognitive functions such as language, attention and emotional control [1]. In particular, Purkinje cells (as principal neurons), which are located between the molecular and granular cell layers of the cerebellum, receive inputs from vestibular, sensory and motor structures to allow for spatially and temporally exact movement [2]. It has been found that cerebellar damage produces disorders in equilibrium, fine movement, posture and motor learning in humans [3]. With regard to ischemia and reperfusion injury in these disorders, experimental rodent models of transient whole-body ischemia and reperfusion injury induced by cardiac arrest and the return of spontaneous circulation (CA/RoSC) have been established to investigate whole-brain damage, including cerebellar damage. In particular, these models have been used to investigate the mechanisms of injury, protection and/or therapy in the cerebellum following CA/RoSC (transient whole-body ischemia) [4,5]. It has been reported that Purkinje cells are killed following transient global brain ischemia induced by CA/RoSC; however, the exact mechanism of Purkinje cell death is still unclear.

Researchers have reported that several antiepileptic drugs have beneficial effects against various kinds of central nervous system (CNS) insults, such as ischemic stroke, intracerebral hemorrhage and traumatic brain injury [6,7,8,9]. In addition, several studies in humans and experimental models have shown that certain antiepileptic drugs (i.e., valproic acid, oxcarbazepine and topiramate) exhibit antioxidant effects by modulating antioxidant enzymatic activities [10]. Among the antiepileptic drugs, oxcarbazepine (OXC; a carbamazepine analog), is one of the most commonly used anticonvulsant compounds in epilepsy treatment [11]. It has been reported that the principal mechanism of antiepileptic activity of OXC involves a blockage of voltage-gated sodium channels (VGSC) [12], and it has been suggested that this mechanism contributes to attenuating cerebral ischemic injury [13]. In addition, OXC exerts beneficial effects by preventing the release of extracellular glutamate and the alteration of recurrent depolarization [14]. Recently, the neuroprotective effects of OXC against brain injury induced by ischemia and reperfusion in the forebrain via activating the Nrf2 defense pathway have been demonstrated in a gerbil model of transient forebrain ischemia [15]. However, the exact mechanism of OXC’s neuroprotective effect against ischemic brain damage is still unclear.

Oxidative stress is a major contributor to ischemic brain injury [16,17]. Increasing evidence has demonstrated that ischemia and reperfusion (such as transient ischemia) in the brain trigger oxidative stress, which is caused by the overproduction of reactive oxygen species (ROS) in the injured areas, leading to ischemic damage [18,19]. ROS at high concentrations can be crucial mediators of damage to cellular components, including lipids, proteins and nucleic acids. It has been reported that some VGSC-blocking antiepileptic drugs possess powerful antioxidant properties and exert neuroprotective effects against ischemia and reperfusion brain injury in rodents [20,21,22]. To date, however, the effect of OXC against cerebellar Purkinje cell death induced by CA/RoSC remains unclear. Therefore, in this experiment, we attempted to investigate whether the therapeutic administration of OXC after CA/RoSC in rats saves the cerebellar Purkinje cells from ischemia and reperfusion injury induced by CA/RoSC and whether CA/RoSC-induced oxidative stress is related to the saving of the Purkinje cells from ischemia and reperfusion injury.

## 2. Materials and Methods

### 2.1. Animals and Experimental Protocol

Male Sprague-Dawley rats at ten weeks of age (body weight, 290–305 g) for use in this experiment were obtained from the Experimental Animal Center of Kangwon National University (Chuncheon, Gangwon, Korea). The rats were bred under pathogen-free conditions with adequate temperature (approximately 23 °C) and humidity (approximately 60%). The protocol of this experiment was approved (approval no. KW-200113-1) on 7 February 2020, by the ethical committee of Kangwon University (Institutional Animal Care and Use Committee). The protocol referred to the “Current International Laws and Policies” from the “Guide for the Care and Use of Laboratory Animals” (The National Academies Press, 8th Ed., 2011).

### 2.2. Experimental Groups and OXC Treatment

A total of 175 rats were used for this study and were split into four groups, as follows: (1) sham plus (+) vehicle group (*n* = 14) was given a sham CA/RoSC operation and treated with vehicle (0.3% dimethyl sulfoxide/saline); (2) CA + vehicle group (n = 122) was given a CA/RoSC operation and treated with vehicle; (3) sham + OXC group (n = 14) was given a sham CA/RoSC operation and treated with 200 mg/kg of OXC; and (4) CA+OXC group (n = 25) was given a CA/RoSC operation and treated with 200 mg/kg of OXC.

OXC (200 mg/kg; Sigma-Aldrich, St. Louis, MO, USA) was dissolved in 0.3% dimethyl sulfoxide/saline as a vehicle and administered intraperitoneally at 10 min after CA/RoSC operation.

As shown in Table 1, the rats of each group either naturally died or were sacrificed for this experiment. Seven rats in each sham group were sacrificed at zero hours and 2 days after CA/RoSC to reduce the rat numbers, and seven rats in each CA group were sacrificed at 12 h and one and two days after CA/RoSC.

### 2.3. CA/RoSC Operation

**Figure 1:**
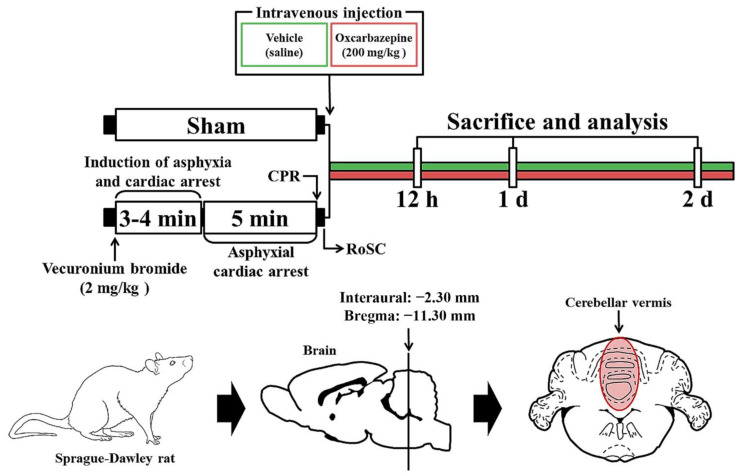
Experimental design and schedule for asphyxial cardiac arrest, cardiopulmonary resuscitation (CPR) and RoSC performed in rats at designated times via the induction of CA, CA, CPR and RoSC. OXC treatment was performed 10 min after RoSC. Sacrifice and analysis were performed at 12 h and one and two days after CA/RoSC.

The CA/RoSC operation was performed according to previously published methods [23,24,25] with minor modifications. Briefly, as shown in Figure 1, the rats were anesthetized with 2–3% isoflurane (in oxygen and nitrous oxide: 33 and 67%, respectively) and ventilated to maintain respiration using a ventilator for rodents (Harvard Apparatus, Holliston, MA, USA). Mean arterial pressure (MAP) was monitored in the left femoral artery using an MP150 blood pressure transducer (BIOPAC system, Goleta, CA, USA). Saturation of percutaneous oxygen (SpO_2_), similar to peripheral oxygen saturation, was monitored using a pulse oximeter (Nonin Medical Inc, Plymouth, MN, USA) connected to the left foot. In addition, the electrocardiogram (ECG) was monitored using an ECG system (GE Healthcare, Milwaukee, WI, USA) which was attached to the four limbs. Body temperature was maintained at 37 ± 0.5 °C during CA/RoSC surgery. In this experiment, CA was induced by asphyxia as follows: After five minutes of stabilization, vecuronium bromide (2 mg/kg; Reyon Pharmaceutical Co., Ltd., Seoul, Korea) was injected via the femoral vein. Immediately, both the anesthesia and mechanical ventilation were stopped, and the endotracheal tube was removed from the ventilator. Asphyxial CA was defined when the MAP was below 25 mmHg and the ECG was isoelectric [24,26]. Usually, asphyxial CA is confirmed approximately three to four minutes after vecuronium bromide injection. After five minutes of CA, cardiopulmonary resuscitation (CPR) was initiated by administering epinephrine (0.005 mg/kg, i.v.; Dai Han Pharm, Seoul, Korea) and sodium bicarbonate (1 mEq/kg, i.v.; Daewon Pham, Seoul, Korea) followed by mechanical chest compression (rate, 300/min) and mechanical ventilation with 100% oxygen until the electrocardiographic activity was shown and MAP reached 60 mmHg [27,28]. Once the rats were spontaneously breathing (usually one hour after RoSC) and hemodynamically stable (usually two hours after CPR), the arterial and venous catheters were cleared. After extubation, the rats were moved to their cages and subcutaneously given isotonic saline (20 mL/kg/d) with 5% dextrose until they could drink and eat by themselves. In this experiment, sham rats were given the same operation except for the CA/RoSC procedure.

### 2.4. Histofluorescence with Fluoro-Jade B (FJB)

To elucidate neuronal death (loss) in the cerebellum following CA/RoSC, Fluoro-Jade B (FJB, a marker for neuronal degeneration) histofluorescence was used as previously described [29]. First, in each group, cerebellar sections containing the vermis were taken from rats (*n* = 7, respectively) at 12 h, one day, and two days after CA/RoSC. The rats were anesthetized with pentobarbital sodium (50 mg/kg; JW Pharmaceutical, Seoul, Korea) and transcardially perfused to collect and rinse their brains with 0.1 M phosphate-buffered saline (PBS, pH 7.4), which were fixed with 4% paraformaldehyde thereafter (in 0.1 M phosphate buffer (PB), pH 7.4). Then, the cerebellums were removed from the skulls and immediately soaked in the same fixative for five hours. To create the sections, thereafter, the cerebellar tissues were saturated to protect the brains from freeze damage with 30% sucrose (in 0.1 M PB, pH 7.4) for eight hours. Finally, 30 µm coronal sections of the cerebellar tissues were collected in a cryostat (Leica, Wetzlar, Germany). For FJB histofluorescence, the cerebellar sections were immersed in 0.0004% FJB (Histochem, Jefferson, AR, USA) on an SW-250S shaker (85 rpm; Gaon Science Co., Bucheon, Korea) for 35 min at room temperature. The sections were washed with distilled water two times (for three minutes each time), and were dried in a dry oven at 45 °C for seven hours. Finally, the cerebellar sections were cleared by dipping in xylene and covered with dibutylphthalate polystyrene xylene (DPX; Fluka, Milwaukee, WI, USA) and a coverglass.

The evaluation of the change in FJB-positive cells (neurons) was performed as follows: Ten cerebellar sections were selected in each rat with a 90 µm interval. FJB-positive cells were observed using a BX53 epifluorescent microscope (Olympus, Tokyo, Japan), which was equipped with a blue excitation light (wavelength from 450 to 490 nm). The digital images of the FJB-positive cells were taken using cellSens Standard image capture software (version, 1.4.1; Olympus, Tokyo, Japan), and the FJB-positive cells were counted in 240 μm^2^ of the Purkinje cell layer in the same area using Image J software (version 1.46; National Institutes of Health, Bethesda, Rockville, MD, USA). The count of FJB-positive cells in each group was conducted by averaging all numbers counted.

### 2.5. Immunohistochemistry

To examine the mechanisms of cell damage or death in the cerebellum after CA/RoSC, according to our method as previously described [29], the cerebellar sections (which were collected as described above) were examined using immunohistochemistry for anti-calbindin D-28kDa (CBD-28k, a marker for cerebellar Purkinje cells), anti-4-hydroxy-2-nonenal (4HNE, an end-product by lipid peroxidation) and anti-SOD1 and -SOD2 (endogenous antioxidant enzymes). In short, the cerebellar sections were blocked with 20% normal goat serum (in 0.05 M PBS) and immunoreacted with primary rabbit anti-CBD-28k (diluted 1:1100; Cell Signaling Technology, Danvers, MA, USA), mouse anti-4HNE (diluted 1:450; Alexis Biochemicals, San Diego, CA, USA), sheep anti-SOD1 (1:1500; Calbiochem, Darmstadt, Germany) and sheep anti-SOD2 (1:1500; Calbiochem) at 4 °C for eight hours. Next, the sections were reacted with biotinylated goat anti-mouse IgG (diluted 1:200; Vector Laboratories Inc., Burlingame, CA, USA), goat anti-rabbit IgG (diluted 1:200; Vector Laboratories Inc.) and goat anti-sheep IgG (diluted 1:200; Vector Laboratories Inc.), then developed using Vectastain ABC (Vector Laboratories Inc., Burlingame, CA, USA). Thereafter, the sections were visualized using 3,3′-diaminobenzidine tetrahydrochloride (DAB; Sigma-Aldrich Co, St Louis, MO, USA) (in 0.1 M Tris-HCl buffer containing 0.1% H_2_O_2_, pH 7.4) under microscopic observation. Once each immunoreaction was defined, the reaction was immediately stopped by washing with 100 mM PBS (pH 7.4), and the sections were dehydrated in graded ethyl alcohol (70, 80, 90, 95 and 100%) and cleared in xylene. Finally, the cerebellar sections were covered with Canada balsam (Kanto Chemical Co., Inc., Tokyo, Japan) and a coverglass.

For the analysis of each immunoreactive (immunostained) structure, ten cerebellar sections were selected in each rat with a 90 µm interval. In order to evaluate the CBD-28k-immunostained cells in all four groups, CBD-28k-immunostained cells were captured in the Purkinje cell layer (the middle layer of the cerebellar cortex) using an AxioM1 light microscope (Carl Zeiss, Göttingen, Germany) with a digital camera (Axiocam, Carl Zeiss, Göttingen, Germany) connected to a PC monitor. The mean number of CBD-28k-immunostained cells was obtained by the method mentioned above. In addition, to evaluate 4HNE, SOD1 and SOD2 immunoreactive structures in all four groups, the staining intensity of 4HNE, SOD1 and SOD2 immunoreactivity was graded as follows: Images of each immunoreactive structure were captured in all layers of the cerebellar vermis using the AxioM1 light microscope (described above). Each image was digitized into an array of 512 × 512 pixels under 20× primary magnification. Each captured image was converted to grayscale (eight bits; 0 to 255, range from black to white) to evaluate the intensity. The optical density of each immunoreactive structure was calculated using Image J software (version 1.46; National Institutes of Health, Bethesda, Rockville, MD, USA). The optical density was presented as relative optical density (ROD; as a %, taking the sham + vehicle group as 100%).

### 2.6. Statistical Analyses

All data obtained here were statistically analyzed using SPSS 18.0 (SPSS, Chicago, IL, USA) and are displayed as means ± standard error of the mean (SEM). Using Kaplan–Meier statistics and the log-rank test, the survival rate was analyzed. MAP and SpO_2_ were compared using one- and two-way analyses of variance (ANOVA). The statistical differences among all four groups were assessed by a post hoc Tukey test. The differences were regarded as statistically significant at a *p*-value under 0.05.

## 3. Results

### 3.1. Physiologic Variables and Survival Rate after CA/RoSC

**Figure 2:**
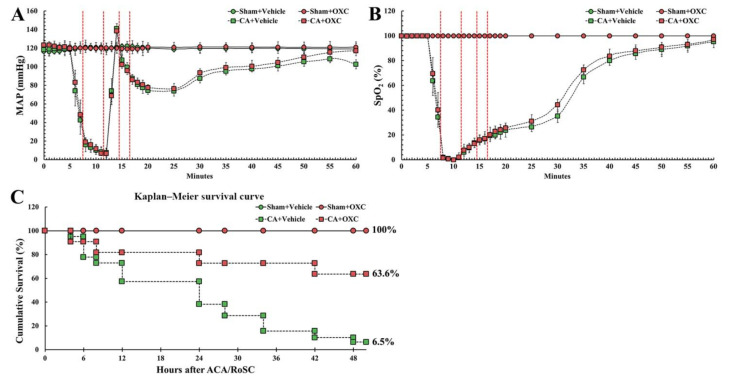
Physiological variables in all four groups. The MAP (**A**) and SpO_2_ levels (**B**) were measured during CA, CPR and RoSC. The bars indicate the means ± SEM. (**C**) The cumulative survival rate of the animals in all four groups after CA/RoSC using Kaplan–Meier analysis two days after CA/RoSC.

CA/RoSC was confirmed by MAP and SpO_2_ in all four groups (sham + vehicle, CA + vehicle, CA/RoSC and CA + OXC groups) (Figure 2A,B). The MAP and SpO_2_ were altered, just as we expected in accordance with the experimental protocol. The survival rate in each group, as shown in Figure 2C, was calculated by the Kaplan–Meier analysis two days after CA/RoSC. The survival rate in the sham + vehicle and sham + OXC groups was 100%; however, the survival rate in the CA + vehicle and CA + OXC groups was 6.5 and 63.6%, respectively.

### 3.2. Salvation of Cerebellar Purkinje Cells by OXC

#### CBD-28k Immunohistochemistry and FJB Histofluorescence

**Figure 3:**
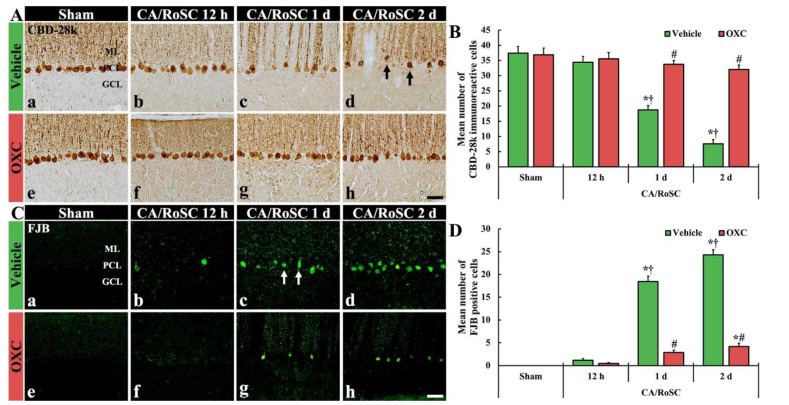
(**A**,**C**) CBD-28k immunohistochemistry (**A**) and FJB histofluorescence (**C**) in the cerebellar vermis of the sham + vehicle (**A**a,**C**a). The CA + vehicle (**A**b–d,**C**b–d), sham + OXC (**A**e,**C**e) and CA + OXC (**A**f–h,**C**f–h) groups at 12 h and one and two days after CA/RoSC. In the CA + vehicle group, the numbers of CBD-28k-Purkinje cells are apparently reduced (arrows) in the Purkinje cell layer (PCL) two days after CA/RoSC, and FJB-cells are apparently increased (arrows) in the PCL at 12 h after CA/PCR. In the CA + OXC group, CBD-28k- and the number of FJB-Purkinje cells are markedly saved and reduced, respectively, two days after CA/RoSC. GCL, granular cell layer; MCL, molecular cell layer. Scale bar = 50 µm. (**B**,**D**). Numbers of CBD-28k-Purkinje cells (**B**) and FJB-Purkinje cells (**D**). The bars determine the means ± SEM (*n* = 7, respectively; *p < 0.05 versus sham, † vehicle group; ^#^ *p* < 0.05 versus CA, † vehicle group).

To examine the damage or death of Purkinje cells, which are located in the middle layer of the cerebellum, after CA/RoSC, the vermis of the cerebellum was examined using immunohistochemistry for CBD-28k (a marker for cerebellar Purkinje cells) and histofluorescence with FJB (a marker for neuronal degeneration) (Figure 3).

In the sham + vehicle group, Purkinje cells were stained with CBD-28k (Figure 3Aa,Ae), and no FJB-positive Purkinje cells (FJB-Purkinje cells) were found (Figure 3Ca,Ce). In the CA + vehicle group, a significant change in the numbers of CBD-28k-immunoreactive Purkinje cells (CBD-28k-Purkinje cells) was not shown at 12 h after CA/RoSC (Figure 3Ab,B); however, at this time, some FJB-Purkinje cells were detected (Figure 3Bb,D). One day after CA/RoSC, the number of CBD-28k-Purkinje cells was significantly decreased (Figure 3Ab,B), and the number of FJB-Purkinje cells was significantly increased (Figure 3Bb,D). Two days after CA/RoSC, the number of CBD-28k-Purkinje cells was more significantly reduced (20.2% of the sham + vehicle group), and the number of FJB-Purkinje cells was more significantly increased (2125.0% of the CA + vehicle group at 12 h after CA/RoSC) (Figure 3Ad,B,Cd,D).

In the sham + OXC group, the distribution of CBD-28k-Purkinje cells was similar to that found in the sham + vehicle group (Figure 3Ae,B), and no FJB-Purkinje cells were found (Figure 3Be,D). In the CA + OXC group, the number of CBD-28k-Purkinje cells was similar to that of the sham + vehicle group at 12 h after CA/RoSC and, at this time, FJB-Purkinje cells were rarely detected (Figure 3Af,B,Cf,D). One day after CA/RoSC, the number of CBD-28k-Purkinje cells was slightly low compared with the sham + vehicle group (Figure 3Ac,B), and the number of FJB-Purkinje cells was significantly decreased compared with that found in the corresponding CA + vehicle group (Figure 3Bc,D). Two days after CA/RoSC, the loss of CBD-28k-Purkinje cells was 85.5% of the sham + vehicle group (Figure 3Ah,B), and the percentage of FJB-Purkinje cells was 17.1% of the CA + vehicle group two days after CA/RoSC (Figure 3Ch,D).

### 3.3. Changes in 4HNE-Immunoreactive Structures by OXC

**Figure 4:**
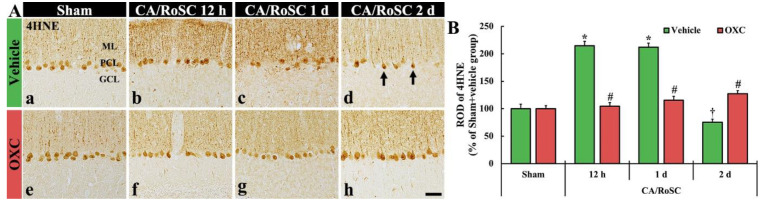
(**A**) 4HNE immunohistochemistry in the cerebellar vermis of the sham + vehicle (**A**a), CA + vehicle (**A**b–d), sham + OXC (Ae) and CA + OXC (**A**f–h) groups at 12 h and one and two days after CA/RoSC. The 4HNE-structures, such as Purkinje cells and their dendrites, are shown in the Purkinje cell layer (PCL) and molecular layer (ML) of the sham + vehicle group. In the CA + vehicle group, 4HNE immunoreactivity is markedly increased at 12 h and one day after CA/RoSC and decreased (arrows) two days after CA/RoSC. In the CA + OXC group, 4HNE immunoreactivity is not significantly altered after CA/RoSC. GCL, granular cell layer. Scale bar = 50 µm. (**B**) Relative optical density (ROD), as a %, compared with the sham + vehicle group (100%). The bars indicate the means ± SEM (*n* = 7, respectively; * *p* < 0.05 versus sham + vehicle group; ^#^ *p* < 0.05 versus CA + vehicle group).

To examine changes in lipid peroxidation in the cerebellar vermis of the four groups, 4HNE (an end-product by lipid peroxidation)-immunoreactive structures (i.e., 4HNE-structures) were examined by immunohistochemistry (Figure 4).

In the sham + vehicle group, the 4HNE-structures, such as Purkinje cell bodies and their dendritic trees, were found in the cerebellar vermis with Purkinje cells comprising the Purkinje cell layer (Figure 4Aa). In the CA + vehicle group, the immunoreactivity of 4HNE-structures was significantly increased at 12 h after CA/RoSC (Figure 4Ab,B) and maintained one day after CA/RoSC (Figure 4Ac,B). Two days after CA/RoSC, the immunoreactivity of 4HNE-structures was significantly decreased (75.4% of the sham + vehicle group) (Figure 4Ad,B).

In the sham + OXC group, the immunoreactivity of 4HNE-structures was similar to that shown in the sham + vehicle group (Figure 4Ae,B). In the CA + OXC group, the immunoreactivity of 4HNE-structures was slightly high at 12 h and one day after CA/RoSC compared with the sham + vehicle group. Two days after CA/RoSC, the immunoreactivity of 4HNE-structures was a little high (127.2% of sham + vehicle group) compared with that evaluated in the sham + vehicle group (Figure 4Af–Ah,B).

### 3.4. Changes in SOD1- and SOD2-Immunoreactive Structures by OXC

**Figure 5:**
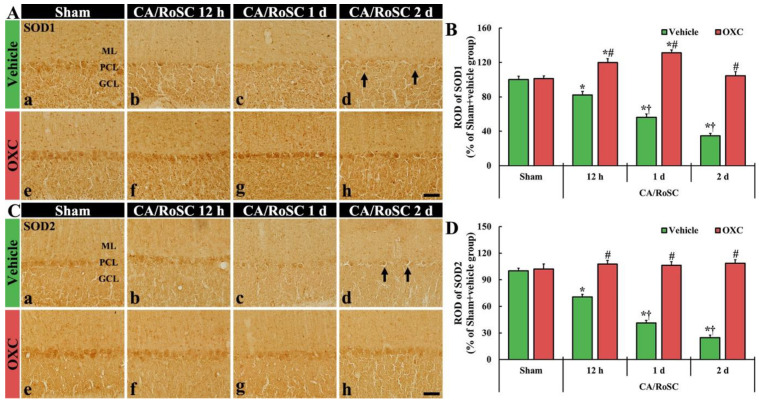
(**A**,**C**) SOD1 and SOD2 immunohistochemistry in the cerebellar vermis of the sham + vehicle (**A**a,**C**a), CA + vehicle (**A**b–d,**C**b–d), sham + OXC (**A**e,**C**e) and CA+OXC (**A**f–h, **C**f–h) groups at 12 h and one and two days after CA/RoSC. In the sham + vehicle group, SOD1- and SOD2-structures are Purkinje cells located in the Purkinje cell layer (PCL). In the CA + vehicle group, SOD1 and SOD2 immunoreactivities are gradually decreased after CA/RoSC and are very low (arrows) two days after CA/RoSC. In the CA + OXC group, SOD1 and SOD2 immunoreactivities are not significantly different from those shown in the sham + vehicle group. GCL, granular cell layer; ML, molecular layer. Scale bar = 50 µm. (**B**,**D**) Relative optical density (ROD), as a % compared with the sham + vehicle group (100%), of SOD1- (**B**) and SOD2- (**D**) structures. The bars indicate the means ± SEM (*n* = 7, respectively; * *p* < 0.05 versus sham + vehicle group; ^#^ *p* < 0.05 versus CA + vehicle group; ^†^ *p* < 0.05 versus CA + OXC group).

To examine changes in SOD1 and SOD2 (as endogenous antioxidant enzymes) in the cerebellar vermis of the four groups, SOD1- and SOD2-immunoreactive structures (i.e., SOD1- and SOD2-structures) were evaluated using immunohistochemistry with SOD1 and SOD2 antibodies (Figure 5).

In the sham + vehicle group, SOD1- and SOD2-structures were principally shown in the Purkinje cells (Figure 5Aa,B,Ca,D). In the CA + vehicle group, the immunoreactivities of SOD1- and SOD2-structures were gradually reduced with time after CA/RoSC, showing 56.3 and 41.2% of SOD1 and SOD2 immunoreactivity one day after CA/RoSC, respectively, and 34.6 and 24.8% two days after CA/RoSC, respectively (Figure 5Ab–d,B,Cb–d,D).

In the sham + OXC group, the immunoreactivity of SOD1 and SOD2 was similar to that of the sham + vehicle group (Figure 5Ae,B,Ce,D). In the CA + OXC group, SOD1 immunoreactivity was significantly increased (119.7 and 131.1%, respectively, of the sham + vehicle group) compared with the sham + vehicle group at 12 h and one day after CA/RoSC. In addition, SOD2 immunoreactivity in the CA + OXC group was slightly increased compared with the sham + vehicle group at 12 h and one day after CA/RoSC (Figure 5Af–h,B,Cf–h,D).

## 4. Discussion

In the brain, specific areas or structures are selectively injured after a transient ischemic episode (ischemia and reperfusion injury), and the topographical heterogeneity of ischemic damage has been described as “selective vulnerability of the brain” [30,31]. It has been reported that the cerebellum, which is involved in the maintenance of balance and posture, the coordination of voluntary movements, motor learning and cognitive functions, is sensitive to ischemic insults indicated by the damage or death (loss) of Purkinje cells [4,32,33]. Cerebellar Purkinje cells, as principal cells in the cerebellum, play critical roles in motor coordination, cognition and learning [34,35].

The loss of cerebellar Purkinje cells after CA/RoSC can contribute to neurologic dysfunction, including post-hypoxic myoclonus [32,36]. A rat model of asphyxial CA/RoSC developed whole-body ischemia and reperfusion injury [4,5,37]. This technique depends on chemical paralysis and ventilation cessation in order to induce circulatory arrest, and it is reproducible and reliable for the study of whole-body ischemia and reperfusion injuries. Brasko et al. (1995) reported that over 60% of cerebellar Purkinje cells died within seven days following 10 min of CA in rats [26]. Additionally, Welsh et al. (2002) demonstrated that cerebellar Purkinje cells died easily, with up to 47% having died within 10.5 min of whole-brain ischemia in rats [20]. Recently, Quillinan et al. (2015) reported that about 24% of cerebellar Purkinje cells were lost within 24 h after eight minutes of CA in mice [27]. In addition, Cho et al. (2019) showed that, in rats, cerebellar Purkinje cells were positive for CBD-28k antibody and the CBD-28k-Purkinje cells were significantly decreased within two days after asphyxial CA/RoSC, showing that many FJB-Purkinje cells were observed from 12 h to one day after CA/RoSC [38]. In our present study, cerebellar Purkinje cells died quickly after five minutes of CA/RoSC, and most Purkinje cells were lost two days after CA/RoSC. Taken together, it is likely that the death (loss) of cerebellar Purkinje cells following CA/RoSC occurs within two days after the insult.

Recently, Ahn et al. (2019) reported that pre- and post-treatment with OXC exerted neuroprotective effects in the hippocampus of a gerbil model of transient global cerebral ischemia, showing that OXC saved hippocampal pyramidal neurons from ischemic injury [39]. To date, few studies have reported the therapeutic effects of OXC on ischemic injury in the cerebellum induced by CA/RoSC. Here, we investigated the therapeutic effects of OXC on cerebellar Purkinje cell death following asphyxial CA/RoSC in rats and found that post-treatment with OXC (200 mg/kg) successfully saved cerebellar Purkinje cells from death induced by CA/RoSC (whole-body ischemia and reperfusion). Our results strongly suggest that cerebellar Purkinje cell death induced by CA/RoSC can be protected by OXC post-administration when taken together.

Amassing evidence has shown that ischemia and reperfusion in the brain elicit oxidative stress through the overproduction of ROS, leading to ischemic brain injury. The overproduction of ROS is especially meaningful in the pathogenesis of ischemia and reperfusion injury, particularly after reperfusion [40,41,42]. The overproduction of ROS is able to cause direct damage to cellular components, such as nucleic acids, lipids and proteins in ischemic tissues, leading to cell damage or death [43,44]. Lipid peroxidation is the primary pathophysiological mechanism implicated in ischemic brain injury [43]. In our current study, 4HNE (an end-product of lipid peroxidation) immunoreactivity was significantly increased in the Purkinje cells at 12 h and one day after CA/RoSC. In a gerbil model of ischemia and reperfusion in the forebrain, it was reported that 4HNE immunoreactivity was dramatically enhanced in pyramidal neurons (as the principal neurons) located in the hippocampal CA1 region one day after ischemia was dramatically increased [45]. Collectively, these results suggest that lipid peroxidation by ROS may contribute to cerebellar Purkinje cell death following CA/RoSC in rats, but the mechanism of Purkinje cell death needs more precise study than the present information. In this study, therapeutic treatment with OXC after CA/RoSC significantly decreased 4HNE immunoreactivity in the cerebellar Purkinje cells that suffered an ischemic injury. In this regard, it seems that the attenuation of CA/RoSC-induced lipid peroxidation in cerebellar Purkinje cells by OXC treatment contributes to the protection of the cerebellar Purkinje cells from CA/RoSC injury.

In addition, the mechanisms of neuronal defense against oxidative stress have been reported to involve antioxidant systems [46]. SOD, one of the enzymatic antioxidants present in all oxygen-metabolizing cells, reacts with superoxide radicals to form H_2_O_2_ and plays crucial roles in maintaining low oxidant levels and redox homeostasis in cells and tissues through scavenging the oxidants [47,48]. In the case of ischemia and reperfusion injury in the brain, researchers have demonstrated that SOD is actively involved in neuroprotection after ischemic insults [44,49]. In this regard, it has been reported that various drugs with antioxidant effects can meaningfully protect brain neurons from ischemic insults [50,51]. For example, Cho et al. (2019) showed that melatonin, which is primarily known in animals as a hormone released at night from the pineal gland located in the brain, significantly protected cerebellar Purkinje cells from ischemia and reperfusion injury induced by CA/RoSC, thereby demonstrating that melatonin treatment dramatically enhanced SOD1 and SOD2 expression using Western blot and immunohistochemical approaches [38]. In our present study, the immunoreactivities of SOD1 and SOD2 were detected in the cerebellar Purkinje cells of the sham + vehicle group, and in the CA + vehicle group, the two immunoreactivities were gradually and significantly decreased after CA/RoSC. However, in the CA + OXC group, the SOD1 immunoreactivity was apparently enhanced at 12 h and one day after CA/RoSC; SOD2 immunoreactivity was slightly increased and was maintained after CA/RoSC. These results collectively suggest that the enhancement or maintenance of antioxidant activity in ischemic brain cells or tissues can be beneficial for neuronal survival after ischemia and reperfusion injury.

## 5. Conclusions

In brief, the therapeutic administration of OXC after CA/RoSC significantly increased the survival rate of the animals (≥63.6%) compared with vehicle-treated rats with CA/RoSC (6.5%), showing that OXC treatment saved cerebellar Purkinje cells (principal neurons) from ischemia and reperfusion injury induced by CA/RoSC. In the ischemic Purkinje cells, 4HNE expression was significantly enhanced, and SOD expressions were markedly reduced, but the OXC treatment for rats with CA/RoSC inhibited the increase in 4HNE and enhanced or maintained SOD expression in the ischemic Purkinje cells. Taken together, we suggest that OXC can be developed as a therapeutic approach for injuries induced by ischemia and reperfusion, including injury by CA/RoSC.

## Figures and Tables

**Table 1:** Actual numbers of surviving animals in each group according to time after CA/RoSC.

	Hours after CA/RoSC									
Groups	0	4	6	8	12	24	28	34	42	48
**Sham + vehicle**	14 ^a^	7	7	7	7	7	7	7	7	7 ^a^
**Sham + OXC**	14 ^a^	7	7	7	7	7	7	7	7	7 ^a^
**CA + vehicle**	122	116 ^b^	95 ^c^	89 ^b^	70 ^a,d^	44 ^a,e^	31 ^b^	17 ^f^	11 ^b^	7 ^a,g^
**CA + OXC**	25	25	24 ^h^	23 ^h^	23 ^a^	15 ^a,h^	8	8	7	7^a^

^a^ Seven animals were sacrificed and excluded in the calculation of cumulative survival rate; ^b^ Six animals were dead; ^c^ 21 animals were dead; ^d^ 19 animals were dead; ^e^ 12 animals were dead; ^f^ 14 animals were dead; ^g^ two animals were dead; ^h^ one animal was dead.

## Data Availability

The data are contained within the article.
